# Diameters of Arteries Supplying Horseshoe Kidneys and the Level They Branch off Their Parental Vessels: A CT-Angiographic Study

**DOI:** 10.3390/jcm8040464

**Published:** 2019-04-05

**Authors:** Marcin Majos, Agata Majos, Michał Polguj, Konrad Szymczyk, Jakub Chrostowski, Ludomir Stefańczyk

**Affiliations:** 1 Department of Radiology, Barlicki University Hospital, Medical University of Łódź, Kopcińskiego Str. 22, 90-153 Łódź, Poland; marcin.majos@stud.umed.lodz.pl (M.M.); konrad.oskar@gmail.com (K.S.); ludomir.stefanczyk@umed.lodz.pl (L.S.); 2 Department of Radiological and Isotopic Diagnosis and Therapy, Medical University of Łódź, ul. Pomorska 251, 92-213 Łódź, Poland; agata.majos@umed.lodz.pl (A.M.); jchrostowski90@gmail.com (J.C.); 3 Department of Angiology, Interfaculty Chair of Anatomy and Histology, Medical University of Łódź, Żeligowskiego 7/9, 90-752 Łódź, Poland

**Keywords:** renal artery, multiple renal arteries, horseshoe kidney, computed tomography angiography

## Abstract

*Background*: The most common renal fusion anomaly is horseshoe kidney (HSK), a condition associated with variable arterial blood supply. The aim of this study was to determine whether the height of origin of the renal artery determines its diameter and whether it differs from the mean diameter of the renal arteries of normal kidneys (NK). *Methods*: Computer tomography angiography (CTA) studies of 336 patients (88 HSK and 248 NK) were obtained in a search of renal arteries; these were than classified into four groups according to height of its origin: Group I, branching from the aorta superior to the inferior mesenteric artery (IMA); Group II, branching from the aorta below the IMA; Group III, branching from the iliac artery; and Group IV, originating from the internal and external iliac artery. *Results*: The HSK group included 398 arteries (mean diameter 4.30 mm) and the NK group 598 (5.52 mm) (*p* < 0.0001). In the HSK group, the mean diameters according to groups were: Group I, 4.54 mm; Group II, 4.28 mm; Group III, 3.41 mm; and Group IV, 3.43 mm. Statistically significant differences were found between arteries originating from the aorta and arteries branching from the iliac arteries (*p* < 0.0001). In the NK group, the corresponding values were: Group I, 5.53 mm; and Group II, 4.45 mm. The number of arteries supplying the HSK wider than 3.0 mm were: Group I, 83.0%; Group II, 82.4%; Group III, 68.4%; and Group IV, 66.6%. *Conclusions*: The renal arteries of the HSK branch from their parental vessels at lower levels and have smaller diameters than those of NK.

## 1. Background

Horseshoe kidneys are one of the most common developmental renal malformations [1,2,3]. Their atypical morphological structure seems to also be associated with the considerable variability of the vascular system [4,5].

For normal kidneys, the vascular system is a source of numerous complications during surgical procedures involving abdominal region, such as kidney transplantation, implantation of vessel stents, kidney stone surgery or aortic aneurysm management [6,7,8]. Although procedures involving horseshoe kidneys are not as well described, their importance is likely to increase as they are affected by similar diseases as typical kidneys [9,10,11,12].

Horseshoe kidneys are more likely to be supplied by additional renal arteries than normal kidneys [5]. Moreover, the arteries for horseshoe kidneys show markedly greater variability with regard to the levels at which they branch off from their parental vessels [1,2,5]. However, to the best of our knowledge, no previous study has compared the diameter of the arteries supplying horseshoe kidneys and normal kidneys with the aim of verifying whether this parameter depends on the level at which they branch off from their parental vessels.

## 2. Methods

A retrospective analysis was performed on the images stored at the PACS archiving system at the Department of Radiology, University Clinical Hospital No. 1 in Łódź. This group of images were obtained from consecutive patients in whom computed tomography (CT) angiography (GE Healthcare, Milwaukee, WI, USA; kV 120, mA 10, mAs dynamic) of the abdominal aorta demonstrated horseshoe kidney (2006–2018) or normal kidney (2016–2017). The protocol of the study was approved by the Local Bioethics Committee of the Medical University of Łódź, Poland (decision no. RNN/132/17/KE).

CT angiography investigation was performed with a GE Light Speed 64 VCT scanner (0.625-mm layer width and a 0.6-mm pitch), after intravenous administration of 80–100 mL of Ultravist 370 contrast agent (BAYER Schering Pharma AG, Berlin, Germany) with an automatic syringe at a flow rate of 4.5 mL/s. All axial, sagittal and coronal CT-angiographic images were evaluated at GE Advantage Workstation (AW 4.0, GE Healthcare).

The inclusion criterion was the presence of a single horseshoe kidney (horseshoe kidney group) or two normally kidneys (control group). The exclusion criteria included the presence of kidney transplantation or renal artery angioplasty, complete or partial resection of the kidney, poor quality or inadequate CT angiograpy (insufficient contrast enhancement, lack of kidney components on the image, motor artifacts or other artifacts hindering comprehensive evaluation of renal arteries, e.g., barium contrast in the intestines or metallic hardware in the spine).

**Figure 1:**
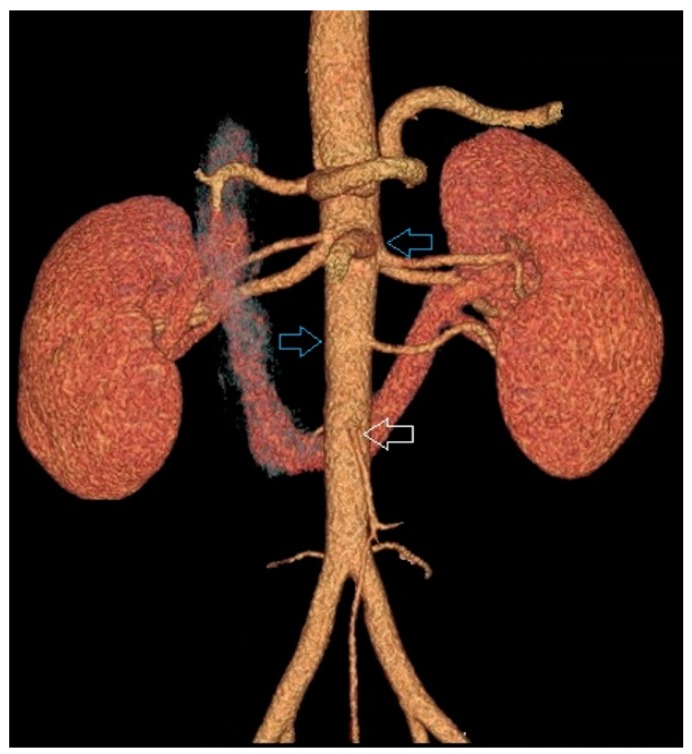
Example of two separated kidneys both supplied by supernumenery renal arteries branching from the aorta above the inferior mesenteric artery.

**Figure 2:**
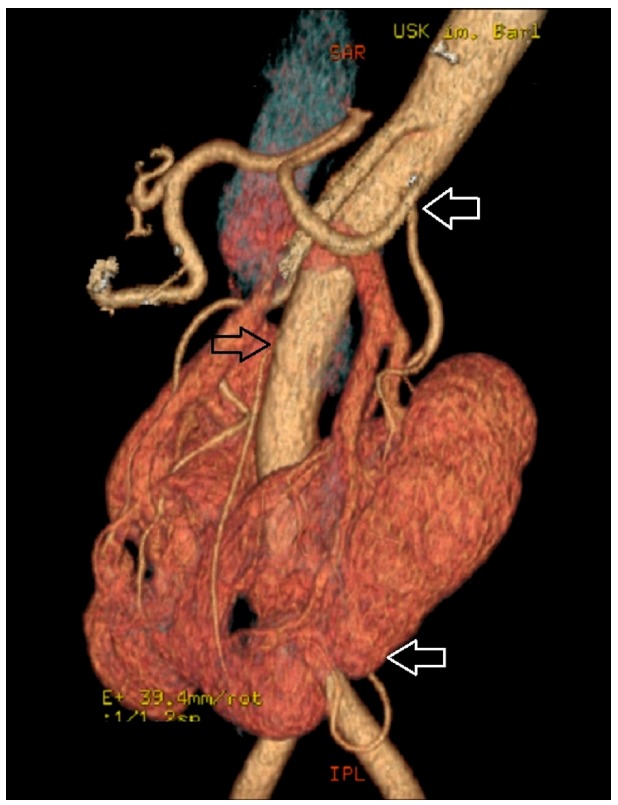
Horseshoe kidney supplied by supernumerary arteries, the first originating from the aorta above the inferior mesenteric artery and the second branching from the left common iliac artery.

The control group comprised 248 subjects (122 women and 126 men) with two normal kidneys (NK). Mean age of the controls was 66.4 ± 15.01 years (between 24 and 94 years) (Figure 1). The horseshoe kidney (HSK) group included 88 persons (34 women and 54 men). The mean age of the study group was 66.0 ± 17.4 years (ranging from 15 to 96 years) (Figure 2). 

**Figure 3:**
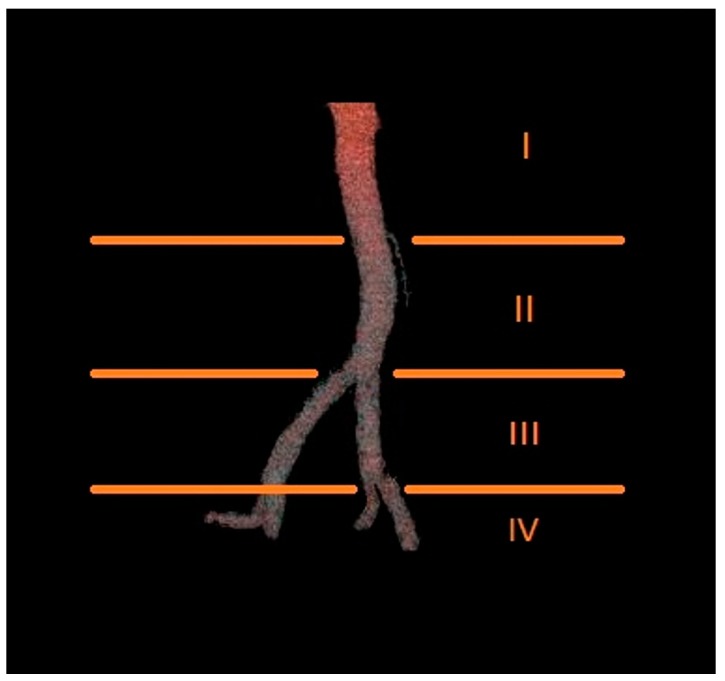
Diagram presenting the proposed levels of origin of renal arteries.

The number of renal arteries supplying each kidney was determined, along with the level at which they branched off from the aorta or iliac arteries. Based on this criterion, the origin of each renal artery was classified into one of four groups: Group I, above the origin of the inferior mesenteric artery; Group II, between the origin of the inferior mesenteric artery and the aortic bifurcation; Group III, between the aortic bifurcation and the bifurcation of the common iliac artery; and Group IV, below the bifurcation of the common iliac artery (Figure 3).

The diameter of each renal artery was then measured 15 mm distally from its parental vessel. Based on this criterion, renal arteries were classified to one of four groups: those with diameters below 4.0 mm; those ranging from 4.0 mm to 5.7 mm; those from 5.7 mm to 7.4 mm; and those above 7.4 mm. These cut-off values were derived from the median value and standard deviation of renal artery diameter in the group of NK. The diameters of the vessels were measured only on axial images, whereas coronal and sagittal images were used to determine the level of branching of the renal arteries. We decided to measure diameters of renal arteries only on axial scans to keep reliability of our study, even though some authors proved that vascular reconstructions are similarly accurate as source data [13,14].

In addition, the diameters of renal arteries were categorized using a clinically relevant cut-off value of 3 mm.

### Statistical Analysis

The quantitative variables were presented as means, standard deviations (SD), medians, minimum and maximum values, and lower and upper quartiles, as appropriate. Before the intergroup comparison of each quantitative variable, the normality of its distribution was verified with the Shapiro–Wilk test. Since the distributions of the study variables in the horseshoe kidney group were not normal, the significance of intergroup differences was verified with the non-parametric Mann–Whitney U-test or Kruskal–Wallis ANOVA with appropriate post hoc tests.

## 3. Results

The study included 398 renal arteries for HSK (mean diameter of 4.30 mm) and 598 renal arteries for NK (mean diameter of 5.52 mm). This difference in renal artery diameter between HSK and NK was statistically significant (*p* < 0.0001). Detailed results are presented in Table 1.

The mean diameters of the renal arteries supplying HSK grouped according to the level at which they branched off from their parental vessels were 4.54 mm for Group I, 4.28 mm for Group II, 3.41 mm for Group III, and 3.43 mm for Group IV. Statistically significant differences in mean renal artery diameter were observed between Group III and Group I (*p* < 0.0001) and between Group III and Group II (*p* < 0.0001); Group IV was not included in the statistical analysis due to its too small size. The mean diameters of renal arteries for NK were 5.53 mm for Group I and 4.45 mm for Group II; the study material did not include arteries from Groups III and IV. Detailed information about the diameters of renal arteries branching from parental vessels at various levels is presented in Table 2.

Furthermore, the renal arteries branching off at various levels were stratified according to their diameters (Table 3). Irrespective of the level at which they branched off from their parental vessels, the majority of renal arteries for HSK were small in diameter (less than 5.7 mm). While Groups I and II included slightly more arteries with diameters between 4.0 mm and 5.7 mm than those smaller than 4.0 mm (41.3% vs. 35.7% and 44.4% vs. 40.7% in Groups I and II, respectively), the vast majority of renal arteries from Group III were less than 4.0 mm in diameter (73.7% vs. 21.1%).

The proportion of HSK renal arteries with diameters greater than 3.0 mm was 83.0% for Group I, 82.4% for Group II and 68.4% for Group I. In turn, up to 91.8% of renal arteries for NK from Group I were more than 3 mm in diameter, and Group II included only one renal artery with a diameter smaller than 3 mm. Irrespective of kidney type and ramification level, renal arteries with diameters greater than 3.0 mm were more frequently found in women than in men. Detailed information about the occurrence of renal arteries with diameters greater than 3.0 mm is presented in Table 4.

## 4. Discussion

Although the topography of individual anatomical structures is essential information for the surgeon, it is even more important in the case of systems or organs, or their parts which show considerable individual variability, such as the arterial system of the kidneys [14]; this is particularly true for the renal arteries for HSK, which display even greater variability than those supplying NK [5]. Furthermore, the development of endovascular procedures, together with other new surgical techniques, and the dramatic expansion of indications for surgical treatment, inter alia those associated with transplantation medicine, require accurate information about the anatomy of arterial and venous supply of the kidneys [15,16,17].

Two classifications of the arterial supply of ectopic kidneys have been proposed thus far. Eisendrath’s classification is based on both the number of renal arteries and the level at which they branch off from their parental vessels [18]. Another classification, proposed by Graves et al., includes the number of renal arteries, the level at which they branch off and their ramification type [19]. However, neither of those classifications applies to the renal arteries originating from the inferior mesenteric artery, internal or external iliac arteries, or median sacral artery. This probably results from the relatively small samples examined in previous studies: Eisendrath’s study only included 132 kidneys. Another potential explanation could be the use of older research techniques, since both Eisendrath’s and Graves’ classification systems were proposed years ago, in 1925 and 1969, respectively. Nowadays, in an era of modern imaging techniques such as digital subtraction angiography, and especially computed tomography and magnetic resonance imaging, all anatomical variants of renal arteries can be identified more comprehensively and accurately [20,21]. Furthermore, renal arteries can be evaluated in many aspects, depending on actual clinical needs. 

However, in contrast to previous publications, the present study made a detailed examination of the arteries supplying HSK with regard to the relationship between their diameter and the level at which they branch off from their parental vessels; this approach is arguably more informative than comparing these vessels with vertebral levels, and forced us to revise our previous analysis by examining the selected population more closely [5]. As noted above, such detailed knowledge of the topography of various anatomical structures is fundamental from the perspective of the surgeon. 

In this study, for NK, only two renal arteries were found to branch off the aorta below the origin of the inferior mesenteric artery, while up to 593 branched off above it. In contrast, 57.79% of renal arteries for HSK branched off the aorta above the inferior mesenteric artery. Thus, nearly half of the renal arteries for HSK (42.21%) branched off the aorta below this level, between the origin of the inferior mesenteric artery and aortic bifurcation (27.14%), or ramified from common iliac arteries (14.32%) or internal and external iliac arteries (0.75%). These results are different to those reported by Crawford et al. [22], which indicate that up to 75% of renal arteries for ectopic kidneys branch off the upper part of the aorta, whereas 25% originate from the lower segment of this vessel. However, those findings were based on a postmortem examination of only 139 kidneys, and the authors did not specify an exact definition for the “upper” and “lower” aorta.

The mean diameter of the arteries supplying the HSK in our material was 4.30 mm, whole those the NK were 5.52 mm in diameter; this difference was statistically significant (*p* < 0.0001). In addition, a significant difference was also observed between the mean diameters of arteries for HSK and NK belonging to Group I, i.e., those branching off the aorta above the origin of the inferior mesenteric artery, (4.54 mm vs. 5.53 mm, *p* < 0.0001).

Furthermore, the mean diameter of the renal arteries branching off the aorta above the origin of the inferior mesenteric artery was not significantly greater than those that branched off below it (*p* = 0.217); however, the mean diameter of renal arteries ramifying from the aorta was significantly greater than that of those branching off the common iliac arteries (*p* < 0.0001). Arteries from Group IV, i.e., the vessels branching off the internal and external iliac arteries, were not included in the statistical analysis due to their small number (*n* = 3); however, the mean diameter of the arteries from Group IV was similar to those in Group III: 3.43 mm and 3.41 mm, respectively. Therefore, it can be concluded that, for HSK, the renal arteries which branched off their parental vessels at a lower level tended to be smaller in diameter. Nevertheless, a statistically significant difference was only found in the diameters of renal arteries branching off the aorta and those ramifying below the aortic bifurcation. 

The present study also examined the diameters of the renal arteries that branched off their parental vessels at different levels. The arteries supplying HSK were divided into four groups based on the median values and SD of the diameters of the renal arteries supplying NK: <4.0 mm, 4.0–5.7 mm, 5.7–7.4 mm and >7.4 mm in diameter. The largest proportion of arteries branching off the aorta were those with diameters between 4.0 mm and 5.7 mm, but those with a diameter <4.0 mm were only slightly less prevalent. In turn, up to 73.7% of renal arteries ramifying from the common iliac arteries had diameters below 4.0 mm. In addition, the renal arteries branching off the aorta above the origin of the inferior mesenteric artery in HSK were significantly more likely to have diameters below 4.0 mm than those for NK ramifying at the same level (35.7% vs. 15.8%). Interestingly, among normal kidneys, only one vessel with a diameter less than 4.0 mm was observed to branch off the aorta below the origin of the inferior mesenteric artery, while the same was observed for up to 40.7% of such small renal arteries for HSK.

The present study also analyzed the diameters of renal arteries supplying HSK from a clinical perspective. As ligation of additional renal arteries with diameters no greater than 3.0 mm has been found to have no significant effect on the degree of renal perfusion [23,24], a 3-mm cut-off value was used for the stratification of renal arteries supplying HSK. Of the vessels branching off the aorta, 83.0% of those above the origin of the inferior mesenteric artery, and 82.4% of below the origin, had diameters greater than 3 mm; in addition, 68.4% those that ramified from the iliac arteries were greater than the cutoff value. These findings are consistent with those published in a computed tomography study of 39 patients by Ichikawa et al., who found that 70% of the renal arteries supplying HSK had diameters greater than 3 mm. Our study adds to those findings, showing that regardless of the level at which they branched off their parental vessels, renal arteries with diameters >3.0 mm were more common in women than in men.

The present study has limitations. The number of patients in analyzed group is relatively small in comparison with typical anatomical studies. In addition, there is a considerable over-representation of men in the HSK group, and the number of renal vessels was not confirmed by any other diagnostic method. 

## 5. Conclusions

To summarize, the renal arteries of HSK typically branched off the aorta above the origin of the inferior mesenteric artery, and were less likely to ramify below the origin of this vessel or from the common iliac arteries and their daughter vessels. The renal arteries of NK also ramified more frequently from the aorta above the inferior mesenteric artery compared to those of the renal arteries of HSK, but they also originated from their parental vessels at different levels: the arteries of HSK were more likely to originate below the level of the inferior mesenteric artery than those of NK.

Irrespective of the level they branched off their parental vessels, the renal arteries of HSK were significantly narrower than those supplying NK. Of the renal arteries for HSK, the vessels branching off the aorta were significantly wider than those originating from common iliac arteries and their daughter vessels.

## Figures and Tables

**Table 1:** The diameters of renal arteries in examined and control group.

	N	Mean (mm)	Median (mm)	Min (mm)	Max (mm)	SD (mm)
Arteries supplying horseshoe kidneys	398	4.30	4.20	0.60	8.50	1.54
Arteries supplying separated kidneys	598	5.52	5.70	1.00	9.80	1.61

**Table 2:** The diameters of renal arteries according to branching level.

	Arteries Supplying Horseshoe Kidneys						Arteries Supplying Separated Kidneys					
Level of Origin of Renal Artery	N	Mean (mm)	Median (mm)	Min (mm)	Max (mm)	SD (mm)	N	Mean (mm)	Median (mm)	Min (mm)	Max (mm)	SD (mm)
I	230	4.54	4.60	0.60	8.50	1.64	596	5.53	5.70	1.00	9.80	1.61
II	108	4.28	4.20	1.40	7.40	1.35	2	4.45	4.45	2.80	6.10	2.33
III	57	3.41	3.30	1.10	6.30	1.11	0	0	0	0	0	0
IV	3	3.43	3.20	2.80	4.30	0.78	0	0	0	0	0	0

**Table 3:** Diameter of renal artery with regard to level of origin.

	Arteries Supplying Horseshoe Kidneys				Arteries Supplying Separated Kidneys			
Level of Origin of Renal Artery	<4.0 mm	4.0–5.7 mm	5.7–7.4 mm	>7.4 mm	<4.0 mm	4.0–5.7 mm	5.7–7.4 mm	>7.4 mm
I	82 (35.7%)	95 (41,3%)	38 (16.5%)	15 (6.5%)	94 (15.8%)	201 (33.7%)	234 (39.3%)	67 (11.2%)
II	44 (40,7%)	48 (44,4%)	15 (13.9%)	1 (1%)	1	0	1	0
III	42 (73.7%)	12 (21.1%)	3 (5.3%)	0	0	0	0	0
IV	2	1	0	0	0	0	0	0

**Table 4:** Frequency of renal arteries of diameter higher than 3.0 mm according to level of their origin.

	Arteries Supplying Horseshoe Kidneys			Arteries Supplying Separated Kidneys		
Level of Origin of Renal Artery	Whole Group	♀	♂	Whole Group	♀	♂
I	191 (83.0%)	71 (86.6%)	120 (81.1%)	546 (91.8%)	261 (92.6%)	285 (91.1%)
II	89 (82.4%)	36 (83.7%)	53 (81,5%)	1	0	1
III	39 (68.4%)	12 (70.6%)	28 (70.0%)	0	0	0
IV	2	0	2	0	0	0
